# Rice Paddies Reduce Subsequent Yields of Wheat Due to Physical and Chemical Soil Constraints

**DOI:** 10.3389/fpls.2022.959784

**Published:** 2022-07-22

**Authors:** Rui Yang, Zhuangzhi Wang, Shah Fahad, Shiying Geng, Chengxiang Zhang, Matthew Tom Harrison, Muhammad Adnan, Shah Saud, Meixue Zhou, Ke Liu, Xiaoyan Wang

**Affiliations:** ^1^Hubei Collaborative Innovation Center for Grain Industry, School of Agriculture, Yangtze University, Jingzhou, China; ^2^Hainan Key Laboratory for Sustainable Utilization of Tropical Bioresources, College of Tropical Crops, Hainan University, Haikou, China; ^3^Department of Agronomy, Faculty of Agricultural Sciences, The University of Haripur, Haripur, Pakistan; ^4^College of Agronomy and Biotechnology, China Agricultural University, Beijing, China; ^5^Tasmanian Institute of Agriculture, University of Tasmania, Launceston, TAS, Australia; ^6^Department of Agriculture, University of Swabi, Swabi, Pakistan; ^7^College of Life Science, Linyi University, Linyi, China

**Keywords:** rotation system, wheat, soil physical properties, soil chemical properties, roots

## Abstract

Yields of wheat crops that succeed rice paddy crops are generally low. To date, it has been unclear whether such low yields were due to rice paddies altering soil physical or mineral characteristics, or both. To investigate this quandary, we conducted field experiments in the Jianghan Plain to analyze differences in the spatial distribution of wheat roots between rice-wheat rotation (RW) and dryland-wheat rotations (DW) using a range of nitrogen treatments. Dryland wheat crops were preceded by either dryland soybean or corn in the prior summer. Biomass of wheat crops in RW systems was significantly lower than that of DW for all N fertilizer treatments, although optimal nitrogen management resulted in comparable wheat yields in both DW and RW. Soil saturated water capacity and non-capillary porosity were higher in DW than RW, whereas soil bulk density was higher in RW. Soil available nitrogen and organic matter were higher in DW than RW irrespective of N application, while soil available P and K were higher under RW both at anthesis and post-harvest stages. At anthesis, root length percentage (RLP) was more concentrated in surface layers (0–20 cm) in RW, whereas at 20–40 cm and 40–60 cm, RLP was higher in DW than RW for all N treatments. At maturity, RLP were ranked 0–20 > 20–40 > 40–60 cm under both cropping systems irrespective of N fertilization. Root length percentage and soil chemical properties at 0–20 cm were positively correlated (*r* = 0.79 at anthesis, *r* = 0.68 at post-harvest) with soil available P, while available N (*r* = −0.59) and soil organic matter (*r* = −0.39) were negatively correlated with RLP at anthesis. Nitrogen applied at 180 kg ha^−1^ in three unform amounts of 60 kg N ha^−1^ at sowing, wintering and jointing resulted in higher yields than other treatments for both cropping systems. Overall, our results suggest that flooding of rice paddies increased bulk density and reduced available nitrogen, inhibiting the growth and yield of subsequent wheat crops relative to rainfed corn or soybean crops.

## Introduction

The substantial increase in the area of crop rotation is driven by increasing population demands for food, feed, and fuel and the development of short-duration cultivars (Timsina and Connor, 2001; Das et al., 2018). Crop rotation is an efficient planting mode to improve land productivity (Zhao et al., 2020) and resource utilization efficiency (Xin and Tao, 2019), which can alleviate many ecological and environmental problems, but it still has some negative effects. Long-term high-load crop rotation will cause problems, such as compaction of the soil sublayer (Götze et al., 2016), soil degradation (Rubio et al., 2021), and decreased soil water storage capacity (Sandhu et al., 2020). Land production conditions directly affect the security of food production and supply, so the soil quality and sustainability of crop rotation systems are long-term concerns.

Paddy-upland rotation is the main planting system in China, India, Pakistan, and other Asian countries with large populations (Yu et al., 2018; Zhou et al., 2022). When wheat is the main upland crop, the crop rotation is mainly summer rice-winter wheat, which is mainly distributed in the Yangtze River Basin in China, mainly concentrated in the plain area of 28°–35°N (Jiang et al., 2022). The dryland crop rotation is dominated by summer corn-winter wheat, summer soybean-winter wheat, mainly distributed in the North China Plain at 32°–35°N (Zhang and Lu, 2019). Jianghan Plain is one of the main grain production bases in Hubei Province and an important part of the wheat area in the Yangtze River Basin. The production of wheat in this region includes both paddy-upland rotation and upland-upland rotation (Yang et al., 2021).

A distinctive feature of the paddy-upland rotation is that the alternation of water and drought in the crop system leads to the alternation of drying and wetting seasons in the soil system. The strong transformation of water and heat conditions causes the alternation of soil’s physical, chemical, and biological properties between different crop seasons, which makes the paddy-upland rotations significantly different from the dryland crop rotation in terms of the material cycle, energy flow, and transformation (Zhou et al., 2014). Deterioration of soil physical quality is a widespread challenge in paddy-upland rotation. In the paddy-upland rotation, rice production requires wet cultivation to break the soil into particles, which is convenient for rice yield development (So and Ringrose-Voase, 2000), but it damages the physical properties of soil, resulting in shallow plow pans and easily water-saturated soil (Scott et al., 2002). Hard soil reduces the root elongation process, limits root uptake of water and nutrients, and impacts subsequent crops’ growth (Colombi and Keller, 2019).

On the other hand, the soil organic matter (SOM) was more rapidly degraded in an RW cropping system than that in dryland crop–wheat (DW) rotation (Singh and Benbi, 2021). The reason for this may be that paddy-upland rotation is the annual conversion of soil from anaerobic to aerobic and back to anaerobic, resulting in increased losses of soil organic carbon due to frequent tillage, making SOM conservation challenging (Singh and Benbi, 2021). The alternation of wetting and drying in paddy-upland rotation causes changes in the form of N elements. In paddy fields, the soil is dominated by NH_4_^+^ (Pandey et al., 2019), whereas under dryland farming conditions, the NH_4_^+^ in the soil is oxidized to NO_3_^–^ through nitrification (Yang et al., 2021). Nitrification and denitrification make N more susceptible to loss in the soil in the paddy-upland rotation.

Plant roots are an integral part of soil ecosystems and are important carbon sinks and nutrient pools. Many scholars have studied the distribution of roots in the soil and their relationship with soil properties through root length and biomass of roots (Cai et al., 2019; Yang et al., 2021). The distribution pattern of the root system and the physical properties of the soil affect each other. The connection between filled and void spaces is influenced by bulk density and aggregate stability, which determines the root development in the soil (Hao et al., 2020). In addition, root growth, death, and decomposition are also the core links of the soil-nutrient cycle. The litter and exudates of the root system affect the cycle process of soil elements (Tian et al., 2019).

Different from previous studies on the relationship between wheat roots and soil properties, which were carried out under one system of paddy-upland rotation or dryland-cropping rotation, there are few comparative studies on the differences between the two systems in the same region. To this end, this study carried out field experiments in the humid climate of Jianghan Plain to analyze the seasonal differences in soil properties and the spatial distribution of wheat roots between paddy-upland rotation and dryland-cropping rotation of wheat season. The relationships between roots and soil properties were also investigated.

## Materials and Methods

### Field Experimental Details

**FIGURE 1:**
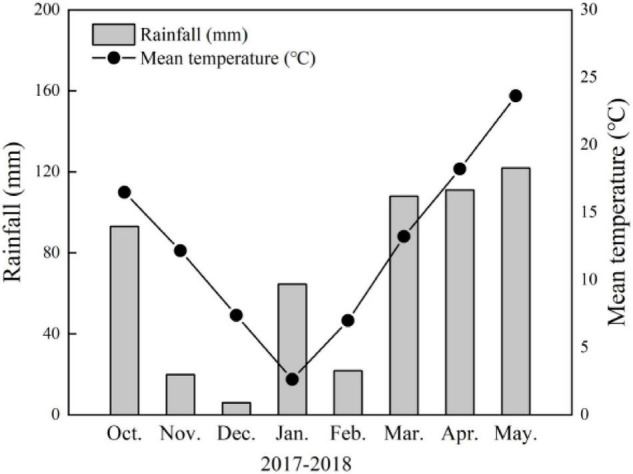
Monthly total rainfall and monthly mean temperatures recorded on-site in 2017–2018 wheat-growing seasons in Jingzhou, Hubei Province, China.

Field experiments were conducted at the experimental farm at Yangtze University (30°36′N, 112°08′E, 34 m asl) in Jingzhou, Hubei Province, China in 2017–2018. The daily mean temperature and rainfall during the wheat-growing season are shown in Figure 1. Meteorological data were collected during each growing season using an automatic weather station adjacent to experimental trials conducted here.

**FIGURE 2:**
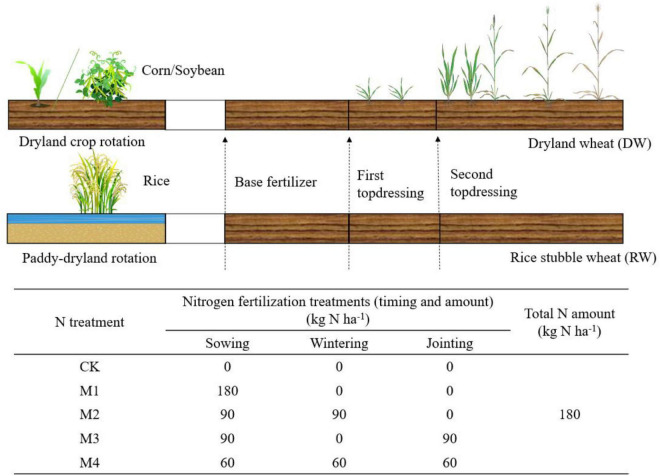
Schematic diagram of crop rotation and nitrogen application timing.

This experiment was conducted with two cropping rotation systems; in both cases, we only focused on the wheat within each rotation (Figure 2):

DW rotation system: The summer crops are dryland crops, mainly corn and soybeans, and winter wheat is DW.
RW rotation system: The summer crop is rice, and winter wheat is RW. This cropping rotation system has a unique wetting and drying cycle soil regime.

Before beginning the experiment, both cropping systems had been used primarily for wheat production for more than 10 years. The tillage soil layer (0–20 cm) of the two cropping systems were as follows: in DW, SOM 11.00 g kg^–1^, available N (AN) 82.03 mg kg^–1^, available P (AP) 15.20 mg kg^–1^, available K (AK) 51.11 mg kg^–1^, and pH 7.8; and in RW, SOM 12.37 g kg^–1^, AN 51.22 mg kg^–1^, AP 12.07 mg kg^–1^, AK 52.74 mg kg^–1^, and pH 7.79.

The treatments were arranged in a split-plot design with a cropping system as the main plots and N treatment as the subplots. N fertilization treatments (timing and amount) were designed in line with local management. N fertilizer was applied in the form of urea (46% N) that was plowed into the soil during sowing and was spread as top-dressing. Each plot is repeated 4 times, arranged in random block groups, with a plot area of 12 m^2^ (2 m × 6 m). The crop rotation and N fertilizer treatments are shown in Figure 2. In the experiment, “Zhengmai 9023” was used as the tested variety, and the planting density was 2.25 million plants ha^–1^. N fertilizer was applied in the form of urea (46% N) that was plowed into the soil during sowing and was spread as top-dressing. Plots were supplied with P (105 kg P_2_O_5_ ha^–1^, calcium superphosphate) and K (105 kg K_2_O ha^–1^ potassium sulfate) fertilizer during the sowing period. We did not need to irrigate during the two growing seasons due to the abundant rainfall. Herbicides, pesticides, and fungicides were sprayed according to standard growing practices to avoid yield loss.

### Grain Yield and Biomass of Wheat

We selected mature plants from a 2-m^2^ harvest area in the middle of each plot to determine the grain yield. Grain moisture was measured using a grain analyzer (Infratec™, Foss, Denmark). Grain yield was adjusted to 13% moisture. The shoot biomass was harvested from twenty mature plants in each plot. Samples were oven-dried at 65°C for at least 48 h until they were a constant weight.

### Root Measurement of Wheat

Root measurements were tested following the protocol described by Liu et al. (2018). Root measurements were made using the CI-600 root growth monitoring system (CID Bio-Science-CI-600, Camas, WA, United States) fitted with a scanner head for collecting images, a laptop computer, and 1-m standard clear soil tubes (50.8-mm internal diameter) with end caps. An auger of the same external diameter as the tube was used to facilitate close tube soil contact. The scanner was inserted into each tube at a depth of 85 cm. Minirhizotron tubes were inserted into the soil of the central sowing line of each plot before sowing. The above-ground part of each tube was covered with thermal insulation foils to prevent light, condensation, and sun warming of the tube. Images were captured at three depths with the aid of an automatic indexing handle equivalent (given the angle of the tube at 45° off vertical) to 0–20, 20–40, and 40–60 cm. Each scan provided a nearly 360° image (21.59 cm × 19.56 cm) with a resolution of 200 dpi. Images were captured at the time of anthesis and after harvest. Root length per sample and tube segments for each plot was calculated from these images using WinRhizotron^®^ software.

### Soil Sampling and Measuring Methods

#### Soil Physical Properties

Bulk density and water-holding capacity were tested following the protocol described by Chen et al. (2019). Soil samples were randomly collected in three replications at depths of 0–20 cm after wheat sowing with cutting cylinders (inner diameter, 50.46 mm; height, 50.00 mm; and volume, 100 cm^3^). The samples were saturated from below by placing them in distilled water. The water was nearly at the level of the soil surface, and it was verified that there was no water that entered the samples from above. The weight of soil water when saturated (W1, g) was measured after the soil core cylinders had been ponding for 24 h. Then, put the soil core cylinders on the sand layer, the weight of water that had been drained by gravity from the saturated samples for 2 h (W2, g). At last, the weight of the cutting cylinders containing dry soil (W3, g) was measured after oven-drying at 105°C for 24 h. Finally wash and record the weight of the hollow cutting cylinder (W4, g). The values were used to calculate the saturated water capacity (kg kg^–1^), capillary-holding capacity (kg kg^–1^), and bulk density (g cm^–3^):


(1) Saturatedwatercapacity(kgkg)-1=W1-W3W3-W4×100%

(2) Capillary-holdingcapacity(kgkg)-1=W2-W3W3-W4×100%

(3) Bulkdensity(gcm)-3=W3-W4100

The total porosity was calculated in undisturbed water-saturated samples of 100 cm^3^ on the assumption that no air was trapped in the pores, and it was validated using dry bulk density and a particle density of 2.65 g cm^–3^ (Danielson and Sutherland, 1986).


(4) Totalporosity(%)=saturatedwatercapacity×bulkdensity×100%

(5) Capillaryporosity(%)=capillary-holdingcapacity×bulkdensity×100%

(6) Non-capillaryporosity(%)=Totalporosity-Capillaryporosity

#### Soil Chemical Properties

Five representative plots were selected for each wheat land use mode at the time of anthesis and after harvest, and 0–20-cm surface mixed soil samples were collected from each plot according to the 5-point sampling method. Soil samples were air-dried, ground, and passed through a 2-mm sieve before chemical analysis. The following soil properties were determined using standard procedures (Bao, 2000): AN, AP, AK, pH in suspension, and SOM. In detail, AN was extracted with 1 mol L^–1^ KCl and measured according to the Alkali Diffusion method. AP was determined spectrophotometrically using the molybdenum blue method. AK was measured by using flame photometry. SOM was measured by the potassium dichromate oxidation method.

### Statistical Analysis

Data were analyzed using a two-way (cropping system and N management) ANOVA with SAS 9.2 (SAS Institute, Cary, NC, United States). Treatment means were compared using the least significant difference (LSD) at *p* < 0.05. The correlation coefficients between soil properties and root length were determined by bivariate correlations with the Pearson coefficients.

## Results

### Grain Yield and Biomass of Wheat

The interactive effects of crop rotation × N treatment were significant for grain yield. Both grain yield and biomass were significantly affected by the crop rotation and N treatment (Table 1). The grain yield observed in DW was significantly higher than the RW cropping system under CK, M1, and M2. But there was a minor yield gap between DW and RW under M4, M5 and did not reach a significant level between the two crop rotations. The grain yield of DW and RW reached the highest grain yield under M5. The biomass was significantly higher in RW than DW at all N fertilization. The biomass of DW was 10.5–108.8% higher than that of RW.

**TABLE 1:** Grain yield and biomass of dryland crop–wheat (DW) and rice–wheat (RW) under different N treatments.

N treatment	Crop rotation	Grain yield (*t* ha^–1^)	Biomass (t ha^–1^)
CK	DW	3.8 aC	8.1 aC
	RW	1.3 bD	3.9 bD
M1	DW	5.0 aB	12.3 aB
	RW	3.9 bC	9.8 bC
M2	DW	5.8 aA	13.3 aAB
	RW	5.2 bB	12.0 bB
M3	DW	5.8 aA	14.2 aAB
	RW	5.7 aB	12.8 bAB
M4	DW	6.4 aA	15.4 aA
	RW	6.6 aA	13.7 bA
ANOVA			
Crop rotation		**	**
N treatment		**	**
Crop rotation × N treatment		**	ns

*The different lower-case letters indicate significant differences (p < 0.05) between DW vs. RW in the same N treatment. The different upper-case letters indicate significant differences (p < 0.05) among the N treatments in the same crop rotation.*

*P < 0.05; **P < 0.01. ns, not significant.

### Soil Physical Properties

**FIGURE 3:**
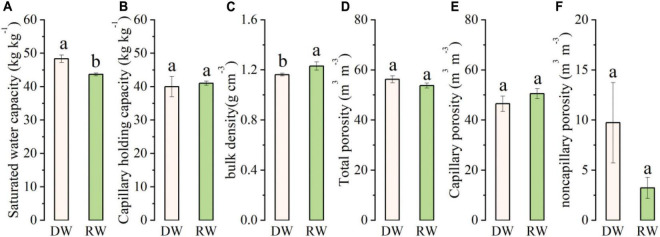
The characteristics of soil physical properties under rice–wheat (RW) and dryland crop–wheat (DW): **(A)** saturated water capacity; **(B)** capillary holding capacity; **(C)** bulk density; **(D)** total porosity; **(E)** capillary porosity; and **(F)** non-capillary porosity. Means and standard error (*n* = 3). Bars with different lower-case letters indicate significant differences at *p* < 0.05.

The soil’s physical properties (soil moisture characteristics, bulk density, and porosity) under wheat season in the RW system and DW system, which averaged over a depth of 0–20 cm, are shown in Figure 3. We chose saturated water capacity and capillary-holding capacity to analyze soil water storage and availability (Figures 3A,B). The results showed that the soil saturated water capacity in DW was significantly higher in DW than RW (*p* < 0.05). However, no significant difference in capillary-holding capacity was found between the two cropping systems. In contrast, the soil bulk density was significantly higher in RW than in DW (Figure 3C). The total soil porosity and capillary soil porosity were at par under both cropping systems; however, non-capillary porosity under the DW system was higher than during wheat season in the RW system, although the difference did not reach a significant level (Figures 3D–F).

### Soil Chemical Properties at Anthesis

**FIGURE 4:**
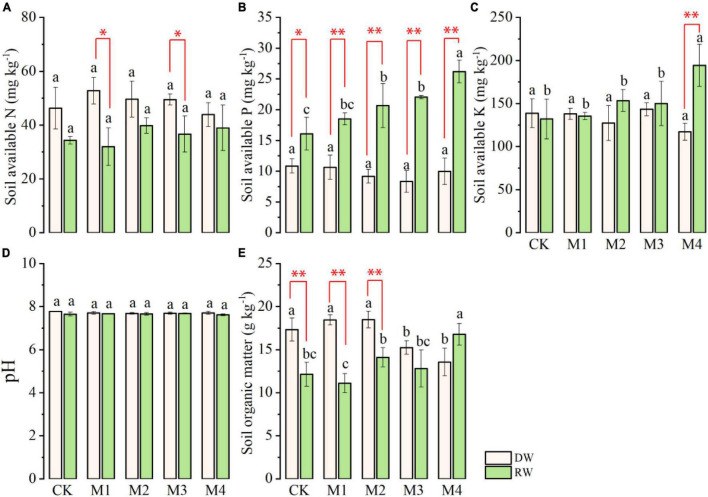
Soil chemical properties (**A**, available nitrogen, AN; **B**, available phosphorus, AP; **C**, available potassium, AK; **D**, pH; **E**, organic matter, SOM) at anthesis stage under dryland crop–wheat (DW) and rice–wheat (RW) cropping systems under different N treatments. The different lower-case letters in the same crop rotation indicate significant differences (*p* < 0.05) among the N treatment. The symbols *, ^**^ indicate significance at *p* < 0.05, 0.01 between DW vs. RW, respectively; blank is not significant.

Analysis of variance indicated that except soil AN and pH, the rest of the soil chemical characteristics (soil AP, AK, and SOM) significantly (Table 2) varied at anthesis under different N treatments in the RW cropping system as shown in Figure 4. However, the performance of N fertilization was at par at all N treatments for all properties except SOM under DW as displayed in Figure 4. The AN observed in DW was significantly higher than the RW cropping system under M1 and CK (Figure 4A). In contrast, the AP was significantly higher in RW than DW at all N fertilization, except in control where both cropping systems showed at par AP. The effect of various N treatments was nonsignificant under DW, whereas, under the RW cropping system, M4 showed maximum AP, which was followed by M3, M2, and M1. However, the lowest AP was observed under the control plot (Figure 4B). Under the RW cropping system, the highest soil AK was observed in M4, which was followed by the rest of N fertilization. There were no intra-treatment differences among M3, M2, M1, and CK for soil AK under RW. The performance of the RW cropping system was significantly better than DW under M4 with respect to AK (Figure 4C). All N treatments are performed at par with each other under both cropping systems for soil PH (Figure 4D). N fertilization significantly affected SOM under both cropping systems (Figure 4E). Maximum SOM was observed in M4, which was followed by M2, M3, and CK; however, the lowest was observed in M2 under RW. In DW, SOM was significantly higher in M2, which was statistically at par with M1 and CK, whereas the lowest SOM was noted in M3 and M4. With respect to SOM, the performance of DW was significantly higher than RW under CK, M1, and M2 as described in Figure 4E.

**TABLE 2:** Analysis of variance (ANOVA) of available N (AN), available P (AP), available K (AK), pH and organic matter (SOM) as affected by crop rotation and N treatment during wheat growing (anthesis) and after wheat harvest.

Index	Crop rotation		N treatment		Crop rotation × N treatment	
	*F*	*P*-value	*F*	*P*-value	*F*	*P*-value
**Anthesis**						
AN	33.23	<0.0001	0.5	0.7342	1.52	0.2353
AP	231.81	<0.0001	4.6	0.0085	7.6	0.0007
AK	10.54	0.004	1.47	0.2473	6.12	0.0022
pH	8.94	0.0072	0.83	0.5224	1.38	0.2755
SOM	46.42	<0.0001	2.48	0.0769	14.37	<0.0001
**Harvest**						
AN	50.6	<0.0001	18.46	<0.0001	7.62	0.0007
AP	286.85	<0.0001	2.81	0.0531	10.52	<0.0001
AK	30.5	<0.0001	3.45	0.0268	14.02	<0.0001
pH	25.66	<0.0001	22.33	<0.0001	2.89	0.0489
SOM	24.09	<0.0001	1.55	0.2253	3.04	0.0416

### Post-harvest Soil Chemical Properties

**FIGURE 5:**
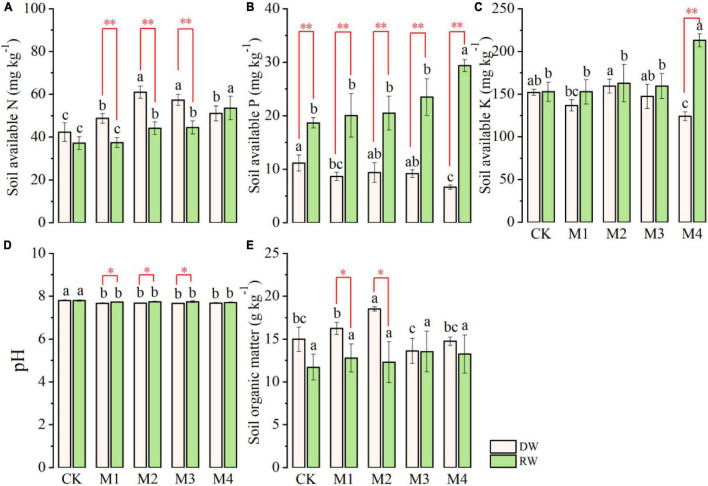
Post-harvest soil chemical properties (**A**, available N, AN; **B**, available P, AP; **C**, available K, AK; **D**, pH; **E**, organic matter, SOM) of dryland crop–wheat (DW) and rice–wheat (RW) under different N treatments. The different lowercase letters in the same crop rotation indicate significant differences (*p* < 0.05) among the N treatment. The symbols *, ^**^ indicate significance at *p* < 0.05, 0.01 between DW vs. RW, respectively; blank is not significant.

Post-harvest soil AN, AP, AK, pH, and organic matter as influenced by cropping systems under different N fertilization treatments are presented in Figure 5. Results regarding soil AN demonstrated that the effect of N fertilization treatments was significant under both DW and RW cropping system as shown in Figure 5A. Generally, AN was comparatively more in DW than RW under all N fertilizers treatments, except CK and M4 where their performance was at par with one another. Under RW, the highest AN was observed in the M4 plot, which was followed by M3 and M2, whereas the lowest soil AN was observed in M1 and CK plots. The intra-treatment performance of CK and M1, and M2 and M3 were at par with one another with respect to soil AN under RW (Figure 5A). The maximum soil AN was recorded under M3 and M4, which was followed by M1 and M4, whereas the lowest AN was observed in CK under the DW cropping system (Figure 5A).

The response of post-harvest soil AP to different N fertilization treatments was also significant (Table 2) under both DW and RW cropping systems (Figure 5B). At each N fertilization treatment (timing and amount), soil AP was significantly higher under RW than DW. Under the RW cropping system, the highest AP was observed under M4, which was followed by the remaining N fertilizers treatments. There were no significant variations among M3, M2, M1, and CK for soil AP under the RW cropping system (Figure 5B). In contrast, under DW, the higher AP was detected in CK fertilizer treatment, which was statistically at par with M1, M2, and M3, whereas the lowest AP was observed in M4 (Figure 5B).

Similarly, post-harvest soil AK was also significantly affected by N fertilization treatments under DW and RW as shown in Figure 5C. The AK was at par under both DW and RW at all N fertilization excluding M4 where the performance of RW was significantly higher than DW with respect to AK. In RW, significantly higher AK was detected under M4, which was followed by the rest of the N fertilizer treatment. There was no intra-significant variation among AK under M3, M2, M1, and CK. In disparity, the AK was maximum under M2, which was statistically comparable to CK and M3 and significantly higher than M4 under the DW cropping system as displayed in Figure 5C.

Nitrogen fertilization did not affect post-harvest soil pH under both RW and DW cropping systems (Figure 5D). However, soil pH was significantly higher in RW than DW under M1, M2, and M3. SOM significantly varied among different N fertilization treatments in DW, whereas in the RW cropping system, it was statistically comparable (Figure 5E). The highest SOM was observed in M2, which was followed by M1, CK, and M4, whereas the lowest SOM was detected in M3 under the DW cropping system. Furthermore, SOM was at par under DW and RW, except M1 and M2 where SOM was significantly higher in DW than RW (Figure 5E).

### Root Length Percentage at Anthesis and Harvest Stage

**FIGURE 6:**
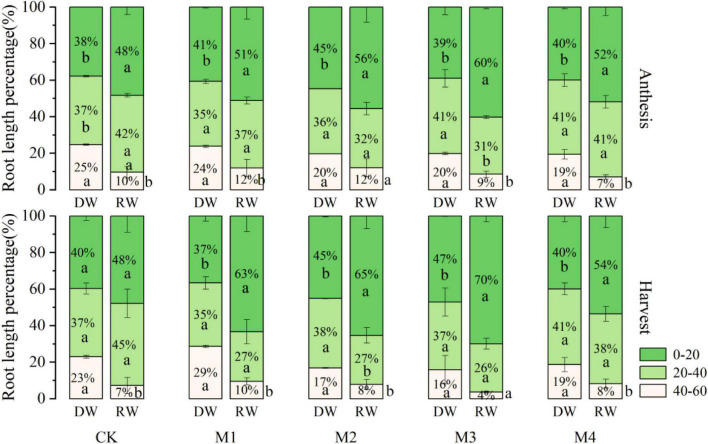
The distribution of wheat roots in 0–60 cm soil, presented by the root length percentage at varying N fertilization under different cropping system at anthesis and harvest stages.

Results concerning the distribution of wheat roots as presented by the root length percentage (RLP) in 0–20, 20–40, and 40–60 cm soil depth, under varying N fertilization in both DW and RW cropping systems at anthesis and harvest stage, are depicted in Figure 6. At the anthesis stage and harvesting, the RLP was mainly distributed on the surface soil layer (0–20 cm), followed by 20–40 cm, and was less distributed in 40–60 cm under both cropping systems regardless of N fertilization. However, the RLP was significantly higher in RW than in the DW cropping system in 0–20 cm depth at all N fertilizers combinations except CK at the anthesis stage. In contrast, under 40–60-cm root zone depth, the RLP was significantly more in DW than RW for all N treatments, except M3 at the anthesis stage and M4 at harvesting. At 0–20 cm depth, the RLP of M3 was higher than that of other N fertilizer treatments in RW at the anthesis stage and harvesting. Under the DW cropping system, the RLP of M2 at the flowering stage was higher than that of other treatments, and the M3 at the harvest stage was the highest. At 20–40 cm depth, the RLP of M4 was higher than that of other N fertilizer treatments, except CK in RW and DW at the anthesis stage and harvesting. At 40–60 cm depth, the RLP of N fertilizer treatments was similar.

### Correlation Analysis

There was a significant positive correlation (*r* = 0.94) between grain yield and biomass in wheat (Table 3). Correlation analysis of wheat grain yield and soil chemical properties at 0–20 cm depth indicated a significantly high positive correlation (*r* = 0.69) between soil AN and grain yield, whereas pH was negatively correlated (*r* = 0.67) with grain yield at harvest stage as shown in Table 3. However, grain yield and biomass were not significantly correlated with soil chemical properties at 0–20 cm at the anthesis stage.

**TABLE 3:** Correlation between wheat growth index [grain yield, biomass, root length percentage (RLP)] and soil chemical properties [available N (AN), available P (AP), available K (AK), pH, and soil organic matter (SOM)] at 0–20 cm.

	Grain yield	Biomass	RLP (0–20 cm)	AN	AP	AK	pH	SOM
**Anthesis**								
Grain yield	1							
Biomass	0.94**							
RLP (0–20 cm)	0.02	−0.06	1					
AN	0.34	0.34	−0.59**	1				
AP	0.02	−0.08	0.79**	−0.52**	1			
AK	0.22	0.14	0.28	0.09	0.69**	1		
pH	−0.08	−0.10	−0.33	0.34	−0.31	−0.14	1	
SOM	0.32	0.26	−0.39*	0.76**	−0.30	0.26	0.22	1
**Harvest**								
Grain yield	1							
Biomass	0.94**	1						
RLP (0–20 cm)	0.05	0.00	1					
AN	0.69**	0.66**	−0.35	1				
AP	−0.05	−0.16	0.68**	−0.28	1			
AK	0.16	0.03	0.29	0.21	0.79**	1		
pH	−0.67**	−0.77**	−0.15	−0.64**	0.33	0.09	1	
SOM	0.35	0.30	−0.46**	0.58**	−0.47**	−0.06	−0.39*	1

*P < 0.05; **P < 0.01.

Correlation analysis of RLP and soil chemical properties at 0–20 cm depth indicated a significantly high positive correlation (*r* = 0.79) between soil AP and RLP, whereas AN (*r* =−0.59) and SOM (*r* =−0.39) were negatively correlated with RLP at anthesis as shown in Table 3. Likewise, there was a strong positive correlation between SOM and AN (*r* = 0.76), whereas post-harvest soil P and N were negatively correlated (*r* =−0.52). The AK was also significantly positively correlated with AP (*r* = 0.69) under 0–20 cm soil depth at the anthesis stage (Table 3). Similarly, the correlation concerning RLP with soil chemical properties at the harvest stage demonstrated that it was significantly and positively correlated with soil P (*r* = 0.68), whereas negatively correlated with SOM (*r* =−0.46) as described in Table 3. The relationship between post-harvest soil N was significantly positive with SOM (*r* = 0.58) and negative with soil pH (*r* =−0.64). Soil AP (*r* =−0.47) and pH (*r* =−0.39) were negatively correlated with post-harvest SOM, whereas AP was positively correlated with AK (Table 3).

## Discussion

Soil health is the ability of soil to act as a dynamic living ecosystem for sustaining humans, animals, and plants (Liu et al., 2020; Tahat et al., 2020). Soil erosion, deforestation, and improper cultivation are the major causes of land degradation across the globe (Ziegler et al., 2011). Moreover, poor agricultural practices further persuade this problem (Lal et al., 2011). Therefore, proper monitoring of soil management practices is crucial for recognizing the variation in soil properties and for maintaining good soil quality for improving soil productivity (Gajda et al., 2020). Paddy-upland rotation is one of the main planting systems in some countries including China (Liu et al., 2022; Yan et al., 2022). We observed that soil saturated water capacity under the DW system was significantly higher (*p* < 0.05) than the RW system. In contrast, the soil bulk density was more in RW than DW, whereas capillary-holding capacity, total soil porosity, and capillary soil porosity were at par under both cropping systems (Figure 3). Our results are in conformity with Saroch et al. (2005). Post-rice wheat yields are generally low in paddy soils that undergo radical changes in physical properties (Yang et al., 2021). It is well-known that the root is affected by external environmental factors. Hard soil reduces the root elongation process, limits root uptake of water and nutrients, and impacts subsequent crops’ growth. Therefore, deterioration of soil physical quality is a widespread challenge in paddy-upland rotation. The improved physical health under DW could be accredited to its improved organic carbon/matter content as shown in Figure 4. According to Libohova et al. (2018), crop rotation effect soil physical conditions, water-use efficiency and stabilize soil temperature by improving ground cover and SOM content. High SOM along with intact root systems maintain stable soil aggregates that further enhance soil water-holding capacity (Six et al., 2006). Porpavai et al. (2011) also stated that rotating legumes with cereal diversify the monocropping system that enhances soil fertility because legume crops are self-sufficient in N supply. Kanwarkamla (2000) observed that the cultivation of legumes in rotation to cereals was comparatively more beneficial in improving soil physical properties compared to independent crops.

The cropping system significantly affected soil chemical attributes, which could be attributed to the differences in composition and quantity of crop residues, and root exudates under different cropping systems (Campbell et al., 1996). Our findings (Figures 4, 5) indicated that AN was comparatively more in DW than RW, whereas soil AP was significantly higher under RW than DW. However, N fertilization did not affect post-harvest soil pH under both RW and DW cropping systems. SOM significantly varied among different N fertilization treatments in DW, whereas in the RW cropping system, it was statistically comparable. Our findings are in line with Rasmussen et al. (2006) who also observed significant variation in extractable P and Zn under different cropping systems. Differences in soil chemical properties under crop rotation have been attributed to long-term fertilizer application within each cropping system (Van Eerd et al., 2014). Dairy farm systems can preserve soil health due to nutrient cycling *via* manure application that includes perennial legumes or grasses. On the contrary, annual grain and vegetables are thoroughly managed and generally do not contain sufficient organic matter (Bender et al., 2015). Therefore, soil health especially, aggregation, porosity, aeration, water holding, organic matter, pH, electrical conductivity, rooting attributes, available nutrients, and microbial biomass and diversity are dependent upon management practices and crop selection (Allen et al., 2011). Most importantly, rooting properties of different crops like depth, branching, turnover rates and exudates secretion affect soil fertility (Rovira, 1969). The improved AP under RW might be due to better mobilization of insoluble P by vigorous root growth and biomass (Congreves et al., 2015). However, the greater variation in SOM under DW can be ascribed to the lower C:N ratio in roots and stubbles that slow down residue decomposition by microbes (Kätterer et al., 2011). Optimum SOM under DW might also likely be due to the fact that residues maintained under DW improve soil microbial activity and aggregate stability (Su et al., 2006). It intensifies soil macro-aggregate fractions that maintain high SOM (Hati et al., 2006). Crop rotation and plant cover significantly affect soil microbial biomass C (Moore et al., 2000) and aggregation and fertility due to the growth of diverse kinds of crops in successive seasons (Balota et al., 2003), and also by an alternation of deep and shallow-rooted crops (Govaerts et al., 2008).

We observed that the RLP was relatively more concentrated on the surface (0–20 cm), followed by 20–40 cm, and were minimum in 40–60 cm under both cropping systems irrespective of N fertilization. However, the RLP was relatively higher in RW than the DW cropping system in 0–20 cm depth at all N fertilizers combinations (Figure 6) as also reported by Govaerts et al. (2008). It may be ascribed to the incorporation of crop biomass into the top soil, which improves root growth (Kulakova et al., 1996). Different crops with varying rooting properties like depth, branching, turnover rates, and exudate secretion affect soil fertility (Rovira, 1969). Root biomass has been reported to contribute more to organic and stable C pools (Congreves et al., 2014). Varying effects of plant types on under-ground root biomass (Ravenek et al., 2014) and root architecture have been reported by Gould et al. (2016), whereas little is known regarding the association of varying plant species and root attributes on soil structural attributes (Pérès et al., 2013).

Although we did not find a significant correlation between the grain yield and the distribution of root length density in the 0–20-cm soil layer (Table 3), the root distribution in different soil environments may have a major impact on the plant’s nutrient uptake and allocation and biomass. We observed that RLP positively correlated with AP (*r* = 0.79), whereas it was negatively correlated with AN (*r* =−0.59) and SOM (*r* =−0.39) at anthesis as shown in Table 3. We observed that the relationship between post-harvest soil N was significantly positive with SOM (*r* = 0.58) and negative with soil pH (*r* =−0.64). Soil AP (*r* =−0.47) and pH (*r* =−0.39) were negatively correlated with post-harvest SOM, whereas AP was positively correlated with AK (Table 3). Very little is known regarding the mutual relationships of RLP with soil chemical properties. For example, positive, weak, or no correlation of root biomass with SOC contents have been documented under diverse cropping systems (Liu et al., 2017). Lee et al. (2017) reported an inverse correlation between root biomass and soil AP in the agriculture and forest echo-system. Cong and Eriksen (2018) found no association between root biomass with any soil chemical properties in unfertilized soils under ryegrass-red clover plantation. Similarly, Chen et al. (2013) have reported a positive correlation between root length and soil AP, whereas Graf et al. (2015) reported an inverse relationship between root length and soil strength. Therefore, our findings suggest that cropping pattern has a substantial influence on soil health and must be properly monitored for plant community performance.

## Conclusion

We found that the yield and biomass of wheat in the RW rotation are inferior to that of the DW rotation (DW) and optimal N management could yield comparable yields of DW and RW. Here, we examined the relationship between soil properties and root growth under different N fertilization between DW and RW cropping systems. Our analysis suggests that soil saturated water capacity and non-capillary porosity were significantly higher in DW than in RW. In contrast, the soil bulk density was significantly higher in RW than in DW. The effect of the cropping system was nonsignificant for soil pH. At the anthesis stage, the RLP was relatively more concentrated on the surface (0–20 cm) in RW than DW in 0–20 cm, whereas at 20–40 and 40–60 cm, it was higher in DW than RW for all N treatments. At harvesting, the RLP was ranked as 0–20 > 20–40 > 40–60 cm under both cropping systems irrespective of N fertilization. Correlation analysis of RLP and soil chemical properties at 0–20 cm depth indicated a significantly high positive correlation (*r* = 0.79 at flowering, and *r* = 0.68 at post-harvest) between soil AP and RLP. N applied at the rate of 180 kg ha^–1^ in three split, 60 kg N ha^–1^ each at sowing, wintering, and jointing performed better than the rest of the N application rates and time under both cropping systems.

## Data Availability Statement

The original contributions presented in this study are included in the article/supplementary material, further inquiries can be directed to the corresponding authors.

## Author Contributions

RY, XW, and SF initiated and designed the research. RY, ZW SG, and CZ performed the experiments. KL, MTH, MA, SS, and MZ revised and edited the manuscript and also provided advice on the experiments. All authors contributed to the article and approved the submitted version.

## Conflict of Interest

The authors declare that the research was conducted in the absence of any commercial or financial relationships that could be construed as a potential conflict of interest.

## Publisher’s Note

All claims expressed in this article are solely those of the authors and do not necessarily represent those of their affiliated organizations, or those of the publisher, the editors and the reviewers. Any product that may be evaluated in this article, or claim that may be made by its manufacturer, is not guaranteed or endorsed by the publisher.
